# Decolonizing global health in an age of fragmentation: reimagining equity for universal health coverage

**DOI:** 10.1093/heapol/czaf109

**Published:** 2025-12-11

**Authors:** Emmanuel Kwasi Afriyie, Ridley Nsioge Mbwoge, Munawar Harun Koray

**Affiliations:** Komfo Anokye Teaching Hospital, Post Office Box 1934, Kumasi, Ashanti Region, Ghana; Kumba Regional Hospital Annex, Southwest Region, Cameroon; Upper West Regional Health Directorate, Post Office Box 132, Wa, Upper West Region, Ghana

**Keywords:** co-production, decolonization, global health equity, health sovereignty, universal health coverage

## Abstract

The global health landscape is undergoing a significant transformation as traditional frameworks of cooperation face fragmentation amid geopolitical tensions. Declining support from Western nations, exemplified by US withdrawal from the World Health Organization and cuts to programs like the President's Emergency Plan for AIDS Relief, has exposed the profound instability of an aid architecture built on colonial dependencies. The COVID-19 pandemic, marked by vaccine nationalism, was a stark litmus test of this systemic failure for low- and middle-income countries (LMICs). This commentary argues that the current geopolitical fragmentation, while a crisis, also presents a critical opportunity to dismantle colonial legacies and reimagine global health equity not as a donor-driven ideal, but as a practice of shared power and sovereignty. We first document the rise of alternative pathways, critically examining China's health diplomacy and India's pharmaceutical disruption, while highlighting robust, LMIC-led initiatives like the African Medicines Agency and local mRNA vaccine production in Rwanda and Thailand. In response to the fractured status quo, we then propose a new global health compact built on four interdependent pillars: (i) Epistemic Justice, valuing local knowledge systems; (ii) Structural Audacity in Financing, such as taxing multinational corporations for reparative funding; (iii) Governance for Agency, ceding decisive power to LMICs; and (iv) Open Knowledge and Innovation, by dismantling restrictive intellectual property regimes. Achieving this decolonized future requires concrete action from all stakeholders. We conclude with a blueprint urging high-income countries to cede power, LMICs to invest in local capacity, funders to provide untied financing, and researchers to practise equitable collaboration. This actionable agenda is the foundation for a truly equitable global health system capable of achieving Universal Health Coverage.

Key messages

- Global health aid is declining due to traditional Western support shrinking. This leaves low- and middle-income countries (LMICs) at risk.
- COVID-19 revealed deep inequality that exists. Rich countries hoarded resources, exposing unfair systems rooted in colonial legacies.
- Alternative paths are emerging to counteract the declining Western support in LMICs. Countries like China, India, and Rwanda are creating new health partnerships and local manufacturing.
- Health systems work better when they include local expertise and traditional practices alongside modern medicine.
- Real equity needs shared power. True progress means LMICs leading decisions, funding, and innovation, not relying on outside charity.

## Introduction

The landscape of global health is undergoing a seismic shift. As traditional architectures of cooperation fragment under geopolitical strain, the urgency of decolonizing health systems becomes undeniable (Abimbola 2021 , Mhazo et al. 2025). National self-interest has always existed in global health, as the responses to H1N1 and Ebola showed, but the current moment is qualitatively different. Scholars of global health governance have charted a decisive departure from the post-Cold War model of multilateral cooperation, which, despite its flaws, was built on a framework of shared technical norms and a nominal commitment to universalism (Kamradt-Scott 2011, Davies and Wenham 2020). Unlike episodic withdrawals or policy divergences of the past, the present fragmentation signifies a systemic unravelling of the normative and institutional frameworks that have shaped global health since its inception (Wenham 2019, Davies and Wenham 2020). Today's fragmentation is more profound; it is not merely about individual nations opting out of specific initiatives, but the deliberate dismantling of the collaborative system itself (Davies and Wenham 2020). It is characterized not just by a resurgence of nationalism but by the systemic structural fragmentation of the multilateral system itself.

Traditional pillars of support, once led by the USA and European nations, are crumbling under the weight of geopolitical realignments, economic pressures, and nationalist turnarounds. Specifically, funding cuts to critical programs like the US President's Emergency Plan for AIDS Relief (PEPFAR; United Nations 2025). This move, coupled with persistent congressional gridlock, further jeopardizes PEPFAR’s life-saving HIV/AIDS programs (United Nations 2025), signals a profound shift in its global health engagement. Additionally, the US’ withdrawal from the World Health Organization (WHO) marks a strategic retrenchment from a domain the USA long dominated as one of the top funders (The White House 2025). This retreat leaves low- and middle-income countries (LMICs) at a precipice: reliant on an unstable and often conditional aid architecture, yet increasingly aware that true health sovereignty cannot be borrowed, bought, or bestowed (Ataguba and Kabaniha 2022).

The COVID-19 pandemic was a litmus test for global solidarity, and the results were damning. While high-income countries (HICs) hoarded vaccines (Tatar et al. 2021), LMICs faced catastrophic shortages, a symptom of deeper structural inequities rooted in colonial legacies that positioned the Global South as perpetual recipients rather than equal partners (MacKellar 2017, Bhambra 2020). In Malawi and Zambia, clinics face medicine stockouts, and healthcare workers migrate en masse, a crisis exacerbated by short-term, crisis-driven funding. This fragmentation is not merely political; it exposes the fragility of a system built on colonial dependencies (MacKellar 2017). Today, fragmentation demands we reimagine equity: not as a donor-driven ideal, but as a practice of shared power.

## The rise of alternative pathways

Yet fragmentation has also unmasked opportunities to redefine health sovereignty. Across the Global South, nations are redefining what health sovereignty means, not by rejecting collaboration, but by reshaping it on their own terms. In this vacuum, alternative models are gaining prominence, though they require critical examination. Major powers are pursuing distinct diplomatic strategies, such as China's health diplomacy, characterized by a ‘no strings attached’ approach and often framed as a counterbalance to Western influence, which has taken on new significance (Lin et al. 2016, Afriyie et al. 2025). Initiatives like supporting the construction of the Africa Centres for Disease Control and Prevention (Africa CDC) headquarters in Ethiopia present a model of infrastructure-driven partnership (Husain and Bloom 2020). While these projects offer tangible benefits in expanded laboratory capacity, commentators note that they are often intertwined with China's strategic self-interest and broader geopolitical goals. This has sparked debates around ‘debt diplomacy’ (Brautigam 2020), where financing is accused of creating unsustainable debt burdens for recipient nations, thereby increasing their long-term geopolitical dependency on China. These valid concerns highlight the need for critical scrutiny of all partnerships, old and new. Meanwhile, India has leveraged its pharmaceutical industry to disrupt monopolies on essential medicines (Chattu et al. 2021, Lavtepatil and Ghosh 2022). By supplying affordable generics and vaccines to LMICs, Indian manufacturers have demonstrated that drug access need not be dictated by Western patent regimes.

Beyond these major powers, LMICs are assertively building sovereignty post-COVID by charting their own course. African nations are pursuing institutional and technical independence, exemplified by the African Medicines Agency, ratified in 2021, which seeks to harmonize regulatory standards and reduce reliance on foreign drug approvals (Hwenda et al. 2022). Rwanda’s collaboration with BioNTech to establish mRNA vaccine manufacturing on the continent is more than a technical achievement; it is a statement of intent (Saied 2023). Similarly, Thailand developed its own mRNA vaccine research and production capabilities through its National Vaccine Institute, aiming to become a regional health security hub (Postigo 2023). These are not alternatives to equity but its reimagination, centring LMIC agency in a fractured world.

## Towards a new global health compact: the heart of reimagination

Reimagining equity requires more than alternative partnerships; it demands a fundamental dismantling of the colonial foundations of global health, from epistemic injustice to inequitable governance (Chaudhuri et al. 2021). This entails building a new compact with a foundational renegotiation of rules, power, and resources based on four interdependent pillars.

## Pillars of a decolonized compact

1. Epistemic Justice: This requires dismantling epistemic colonialism (Lazo-Porras and Penniecook 2023). Global health progress requires valuing diverse forms of knowledge. While Western medicine has made vital contributions, integrating local expertise and traditional practices can strengthen health systems in meaningful ways. Some countries are thoughtfully blending these approaches: Ghana's inclusion of proven herbal remedies in national health policy and Nigeria's emphasis on local research leadership, demonstrates how combining scientific rigour with community wisdom can yield innovative solutions (Asase 2023, Nwaham et al. 2024). By integrating respectful dialogue, traditional knowledge may be neither dismissed nor romanticized, but carefully evaluated alongside biomedical approaches. For example, international partners can share technical expertise while genuinely listening to local perspectives. This balanced integration honours local contexts while maintaining rigorous health standards, creating systems that are both culturally grounded and scientifically sound. When done well, these exchanges enrich global health practice for all involved.
2. Structural Audacity in Financing: Furthermore, it requires structural audacity, where funding mechanisms must shift from donor-controlled models to LMIC-led financing pools. The Global Fund’s emphasis on country ownership is a start, but true equity would involve direct taxation of multinational corporations, particularly pharmaceutical giants, to fund universal health coverage. This aligns with calls for reparative financing that actively rectifies historical extractivism and builds a sustainable, predictable resource base independent of the fluctuating goodwill of HICs (Chaudhuri et al. 2021, Wispelwey et al. 2023).
3. Governance for Agency: The new compact must be rooted in governance that institutionalizes LMIC agency. The principle of subsidiarity that decisions should be made at the most local and appropriate level must become paramount. The WHO’s proposed pandemic accord offers a potential framework, though its success hinges on whether HICs will cede decision-making power (Phelan 2023). True equity requires moving from consultation to leadership, ensuring LMICs have decisive votes in the governance of initiatives that affect their populations.
4. Open Knowledge and Innovation: Additionally, dismantling intellectual property regimes is paramount. The trade-related aspects of the regime’s waiver for COVID-19 vaccines, had won out after years of advocacy. This permitted the Serum Institute of Africa partnership with South Africa to produce COVID-19 vaccines locally, marking a pivotal step towards regional self-reliance. This co-production should be expanded to include all essential medicines. This must be expanded to foster a commons-based approach to research and development, where knowledge is co-produced and shared as a global public good (Gonzales-Gavancho et al. 2025), preventing the monopolization of life-saving technologies. Exemplifying this new narrative is the rise of robust South-South cooperation. This LMIC-led initiative focuses on pooling resources, sharing knowledge, and facilitating technology transfer within the continent, moving decisively away from a model of waiting for Northern permission or support.

## From compact to action: a blueprint for stakeholders

Achieving this future requires concrete actions from all stakeholders. HIC governments must move beyond rhetoric and cede tangible decision-making power in bodies like the WHO, actively support the expansion of Trade-Related Aspects of Intellectual Property Rights waivers, and implement taxes on multinational corporations to fund LMIC-led health initiatives. Conversely, LMIC governments must prioritize strategic investments in local manufacturing and research while thoughtfully integrating traditional knowledge with modern evidence-based medicine. Development partners and funders must shift from restrictive, project-based funding to providing untied, flexible financing directly to local institutions and championing co-production models.

Finally, researchers and academic institutions must practise equitable authorship, prioritize research questions defined by local communities, and commit to sharing data and results openly with their LMIC counterparts. This multifaceted, actionable agenda is the foundation for truly decolonized and equitable global health.

## Author contributions

E.K.A: Conceptualization. E.K.A., R.N.M., and M.H.K.: Investigation, formal analysis, writing—original draft, writing—review & editing. All authors reviewed and approved the final version of the article.

## Reflexivity statement

The authors, a Ghanaian teaching hospital health service administrator (E.K.A.), a Cameroonian health practitioner (R.N.M.), and a Ghanaian healthcare professional (M.H.K.), combine experience with critical perspectives on global health inequities. Their work in resource-constrained African settings informs this decolonial analysis, while acknowledging how their institutional positions may influence certain viewpoints. This reflection emphasizes contextually grounded, equity-focused approaches to health system reform.

## Data Availability

The data underlying this article are derived from publicly available sources, including academic literature, policy documents, and institutional reports. All sources are cited in the reference list. No new data were created or analyzed in this study.
